# Cavity optomechanical spring sensing of single molecules

**DOI:** 10.1038/ncomms12311

**Published:** 2016-07-27

**Authors:** Wenyan Yu, Wei C Jiang, Qiang Lin, Tao Lu

**Affiliations:** 1 Department of Electrical and Computer Engineering, University of Victoria, Victoria, British Columbia, Canada V8P 5C2; 2 Institute of Optics, University of Rochester, Rochester, New York 14627, USA; 3 Department of Electrical and Computer Engineering, University of Rochester, Rochester, New York 14627, USA

## Abstract

Label-free bio-sensing is a critical functionality underlying a variety of health- and security-related applications. Micro-/nano-photonic devices are well suited for this purpose and have emerged as promising platforms in recent years. Here we propose and demonstrate an approach that utilizes the optical spring effect in a high-*Q* coherent optomechanical oscillator to dramatically enhance the sensing resolution by orders of magnitude compared with conventional approaches, allowing us to detect single bovine serum albumin proteins with a molecular weight of 66 kDa at a signal-to-noise ratio of 16.8. The unique optical spring sensing approach opens up a distinctive avenue that not only enables biomolecule sensing and recognition at individual level, but is also of great promise for broad physical sensing applications that rely on sensitive detection of optical cavity resonance shift to probe external physical parameters.

## Abstract (web summary)

Detection of a single nanoparticle or molecule is essential for many applications. Here, Yu *et al.* demonstrate the use of an optical cavity with optomechanical oscillation to detect single bovine serum albumin proteins, with potential for studying mechanical properties and interactions of individual molecules.

## Graphical abstract

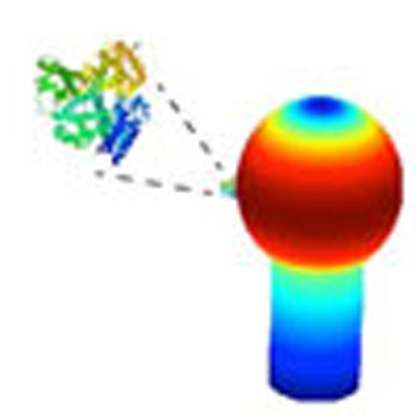

Sensitive detection of a single nanoparticle/molecule is essential for many applications ranging from medical diagnostics, drug discovery, security screening and to environmental science. In the past decades, a variety of approaches have been developed to observe single particles down to molecular scale^1^,^2^, among which optical detection based on high-*Q* microcavities has shown significant advantages for its high sensitivity and label-free operation^3^,^4^,^5^,^6^,^7^,^8^,^9^,^10^,^11^,^12^. Binding of a particle to a high-*Q* optical microcavity perturbs the cavity mode at a resonance wavelength of *λ*_0_, resulting in a cavity resonance shift of *δλ* which in turn changes the cavity transmission. This mechanism underlies the majority of current microcavity sensors, with a sensing resolution dependent critically on the optical quality factor (*Q*)^13^. To date, the highest resolution reported is a resonance shift of (*δλ*/*λ*_0_)=3 × 10^−10^ achieved with an optical *Q* of one hundred million at a visible wavelength in an aqueous environment^8^, which, however, is still larger than that induced by a single protein binding event^6^. Consequently, detection of a single protein molecule down to 1 kDa requires incorporating a plasmonic nanoantenna on the microcavity to enhance the resonance wavelength shift^14^,^15^,^16^,^17^, at the price of a significant reduction of the effective detection area.

On the other hand, the optical wave cycling inside the microcavity is able to produce a radiation pressure that interacts with the mechanical motion of the device. Such optomechanical coupling flourishes in profound physics that has been intensively explored in recent years, particularly in the context of quantum control of mesoscopic mechanical motion^18^,^19^. When the laser wavelength *λ*_l_ is blue detuned to the cavity resonance, the optical wave can efficiently boost the mechanical motion above the threshold of regenerative oscillation^18^,^19^, resulting in highly coherent optomechanical oscillation (OMO) with a narrow mechanical linewidth. Of particular interest is that the optical wave inside the cavity is able to produce an effective mechanical rigidity^19^, leading to an OMO frequency *f*_m_ depending sensitively on the laser-cavity detuning Δ_*λ*_=*λ*_l_−*λ*_0_. Consequently, any tiny perturbation to the cavity resonance wavelength, *δλ*, induced by particle/molecule binding would be readily transferred to the frequency shift, *δf*_m_, of the mechanical motion: 
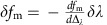
, thus enabling an efficient transduction mechanism to amplify the resonance wavelength sensing.

As the minimal detectable frequency shift of OMO is determined by its linewidth Δ*f*_m_, the minimal detectable optical cavity resonance shift is thus given by 
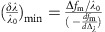
. With a narrow linewidth of coherent OMO and a significant frequency tuning slope 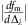 (see below), the intriguing optical spring effect would thus offer an elegant approach for sensitive probing of cavity resonance variation, with a sensing resolution given by (Supplementary Note 1).


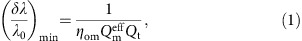


where 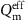 is the effective mechanical *Q* factor of OMO, defined as the ratio between the frequency *f*_m_ and the linewidth Δ*f*_m_ of the OMO: 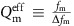. *Q*_t_ is the loaded optical *Q* and *η*_om_ represents the optomechanical transduction factor for sensing whose magnitude depends on laser-cavity detuning, with a value in the order of *η*_om_∼1. Equation (1) shows clearly that the sensing resolution scales not only with the optical *Q* of the cavity as in conventional microcavity sensors, but also with the effective mechanical *Q* of OMO. Consequently, in principle, the proposed cavity optomechanical spring sensing is able to enhance the sensing resolution by about a factor of 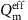 compared with conventional approaches. As we will show below, the effective mechanical *Q* of coherent OMO in our device can reach a value above 10^6^, resulting in a sensing resolution enhanced by orders of magnitude that is sufficient for single-molecule detection.

To date, cavity optomechanics has been widely applied as a sensitive approach for probing mechanical displacement^18^,^19^. However, the magnitude of mechanical motion is not relevant here. Instead, the optically induced frequency shift of the OMO is employed as the information carrier to transduce and to amplify the molecule binding signal. In this sense, we call our approach optical spring sensing. Although the optical spring effect has been known for a decade^19^, we realize its potential for particle and molecule sensing.

As the optomechanical effect is intrinsic to a high-*Q* microcavity, the proposed approach does not rely on any specific external sensing element attached to the device (for example, a plasmonic nanoantenna^15^,^16^,^17^) and is thus capable of fully utilizing the entire effective sensing area offered by a whispering-gallery microcavity which is more than five orders of magnitude larger than that of plasmonic devices. For the same reason, it does not rely on any gain medium and is therefore universal to different material platforms as long as the device has reasonably high optical *Q*. On the other hand, this approach is distinctive from the conventional micro-/nano-mechanical sensing^2^,^20^,^21^,^22^ where particle detection is realized by monitoring the mechanical frequency shift directly induced by the mass change from a particle attaching, which exhibits a minimal detectable mass of 
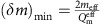
 that relies critically on the motional mass *m*_eff_ and the effective mechanical *Q* of the sensor (see Supplementary Note 2 for detailed discussion). This mechanical sensing principle underlies the optomechanical sensors developed recently^23^,^24^,^25^ while in combination with optical actuation and readout. With significant intrinsic motional masses, these sensors, however, can only detect ∼1-μm-diameter silica beads with a sub-picogram resolution^23^,^24^,^25^. For nanomechanical sensing, achieving single molecule resolution requires extremely tiny mass of employed nanomechanical oscillators^2^,^20^,^21^,^22^.

Here we show that the optical spring sensing principle proposed above is able to dramatically enhance the sensing resolution by orders of magnitude compared with conventional approaches. It allows us to detect single silica nanobeads as small as 11.6 nm in radius and bovine serum albumin (BSA) proteins with a molecular weight of 66 kDa at a signal-to-noise ratio (SNR) of 16.8.

## Results

### OMO of a silica microsphere in buffered solution

**Figure 1:**
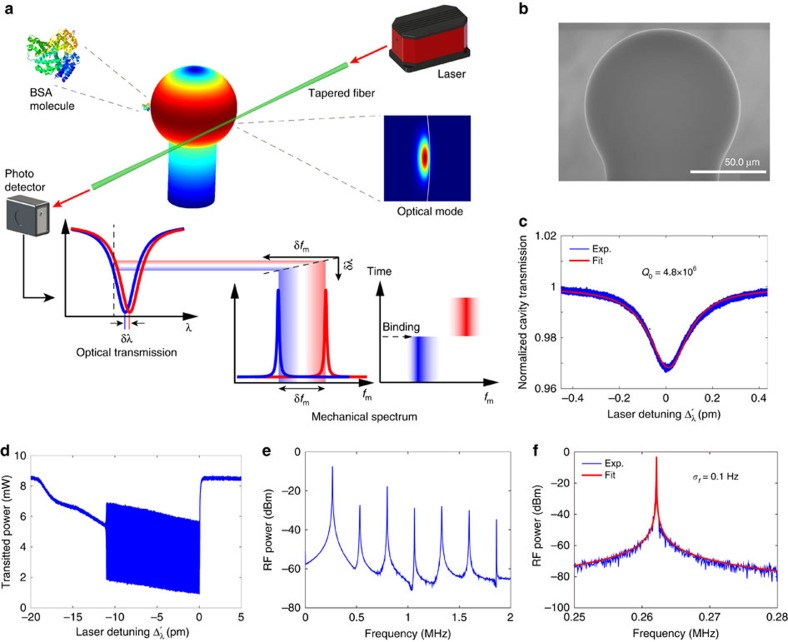
Experiment schematics and device characterization. (**a**) Schematic illustrating the sensing mechanism. A protein molecule bound to an optomechanically oscillating microsphere yields an optical resonance shift *δλ*, which is transduced to a mechanical frequency shift *δf*_m_. The colour map on the microsphere shows the radial breathing mechanical mode simulated by the finite element method. (**b**) A scanning electron microscopic (SEM) image of a fabricated silica microsphere. (**c**) The optical transmission spectrum of the microsphere immersed in DPBS, at a probe laser wavelength of 974 nm, with experimental data in blue and theoretical fitting in red. The input power is maintained low enough to characterize the intrinsic optical property of the device, which exhibits an intrinsic optical *Q* of 4.8 × 10^6^. (**d**) The optical transmission spectrum at an input laser power of 8.5 mW. The coherent OMO was excited with a threshold power of 3.0 mW injected into the cavity. (**e**) An example of the power spectral density of the cavity transmission. The fundamental oscillation frequency is located at 262 kHz, with six high-order harmonics clearly visible on the spectrum. (**f**) The detailed spectrum of the fundamental oscillation tone, with experimental data in blue and theoretical fitting in red. The OMO exhibits a full-width at half maximum of 0.1 Hz, corresponding to an effective mechanical *Q* of 2.6 × 10^6^.

To verify the optical spring sensing principle proposed above, we carried out experiments in a silica microsphere with a diameter of about 100 μm. The device exhibits an intrinsic optical *Q* as high as 4.8 × 10^6^ at a wavelength ∼974 nm in the aqueous environment (Fig. 1c), which is close to the theoretical limit. With such a high optical *Q*, the optical wave inside the microsphere produces a strong radiation pressure that efficiently actuates the radial breathing mechanical motion of the microsphere (Fig. 1a). Consequently, by injecting an optical power of 3.0 mW into the cavity (Fig. 1d), we are able to boost the mechanical mode above the threshold even in the aqueous environment^26^, resulting in coherent OMO at a frequency of 262 kHz with a mechanical linewidth as narrow as 0.1 Hz (Fig. 1f), corresponding to an effective mechanical *Q* of 2.6 × 10^6^. As shown in Fig. 1e, the significant OMO leads to a harmonic comb on the power spectrum of the cavity transmission, a feature of coherent OMO resulting from the nonlinear transduction of the optical cavity^27^,^28^.

### OMO versus laser-cavity detuning

**Figure 2:**
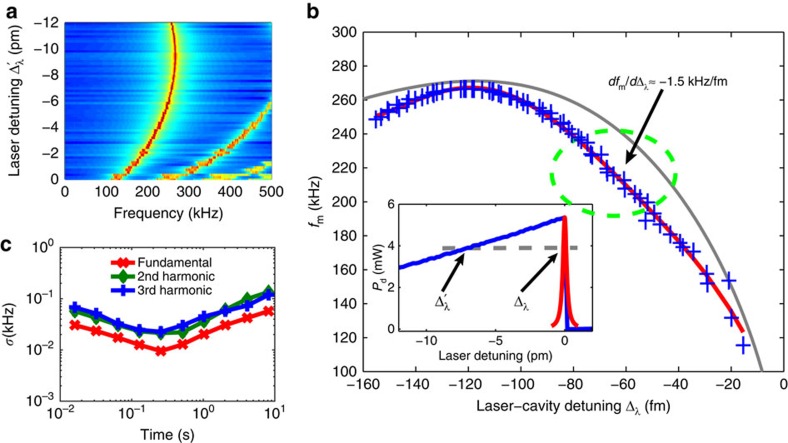
OMO versus laser-cavity detuning. (**a**) Spectrogram of cavity transmitted signal as a function of laser wavelength detuning Δ′_*λ*_ (see Fig. 1d for the meaning of Δ′_*λ*_), showing the detuning-dependent mechanical frequency. The proportional frequency variations at the second and third harmonics are clearly visible. Every spectrum was averaged over five traces acquired continuously. (**b**) The OMO frequency as a function of laser-cavity wavelength detuning. The blue crosses show the experimental data and the grey curve shows the theory. The red curve is a polynomial fitting to the experimental data. The dashed circle indicates the operating regime for the particle and molecule sensing, with a frequency tuning slope of *df*_m_/*d*Δ_*λ*_=−1.5 kHz fm^−1^ at a laser-cavity detuning of Δ_*λ*_=−70 fm. Inset: recorded optical power as a function of laser wavelength detuning. This curve was used to obtain the real laser-cavity wavelength detuning. (**c**) The two-sample Allan deviations of the fundamental, second and third harmonic tones measured in DPBS in the absence of sensing particle, showing a minimum deviation of 9.5 Hz at the fundamental oscillation tone.

In particular, the strong optical spring effect from the optomechanical coupling results in an OMO frequency sensitively dependent on the laser-cavity detuning (Fig. 2a). As shown in Fig. 2b, when the laser-cavity wavelength detuning Δ_*λ*_ decreases from −150 fm, the OMO frequency increases from 247 kHz to a peak value of 267 kHz, and then decreases quickly to about 115 kHz when the laser wavelength is tuned close to the centre of the cavity resonance, with a tuning slope of *df*_m_/*d*Δ_*λ*_≈−1.5 kHz fm^−1^ at a laser-cavity detuning of Δ_*λ*_≈−70 fm. The observed optical spring follows closely the theoretical expectation (grey curve in Fig. 2b; see Supplementary Note 1). The slight discrepancy is likely because the coherent OMO in experiment exhibits a significant amplitude (Fig. 1d) beyond the linear perturbation regime used in the theoretical estimation.

Such an optical spring corresponds to a sensitive optical-to-mechanical frequency transduction, inferring that every 1-fm cavity resonance wavelength shift induced by a particle binding event can be transduced to an OMO frequency change of about 1.5 kHz that is about four orders of magnitude larger than the linewidth of OMO (Fig. 1f). A detailed characterization of the Allan deviation of the OMO frequency (Fig. 2c, see also Methods section) shows a minimum two-sample deviation of 9.5 Hz at the fundamental OMO frequency. This Allan deviation includes all noise in the microcavity, implying a detection resolution of *δλ*/*λ*_0_≈6 × 10^−12^ in the device. This resolution clearly shows the power of the demonstrated approach, which is more than 10^4^ times higher than a conventional microcavity sensor with the same optical quality factor^13^. It is even about 50 times higher than that achieved with an optical *Q* of 10^8^ at a visible wavelength^8^. Note that the harmonics of the OMO vary proportionally with the OMO fundamental frequency (Fig. 2a) and thus can also be applied for particle sensing. Although this does not improve the sensing resolution due to the same SNR of detection, in practice, the larger frequency shifts on the higher order harmonics significantly facilitate the mechanical spectrum analysis (by allowing to use a coarser resolution bandwidth), which reduces considerably the excessive detection noises from consecutive multiple particles skimming by the cavity surface (see discussions in Methods section).

### Detection of silica nanobeads

**Figure 3:**
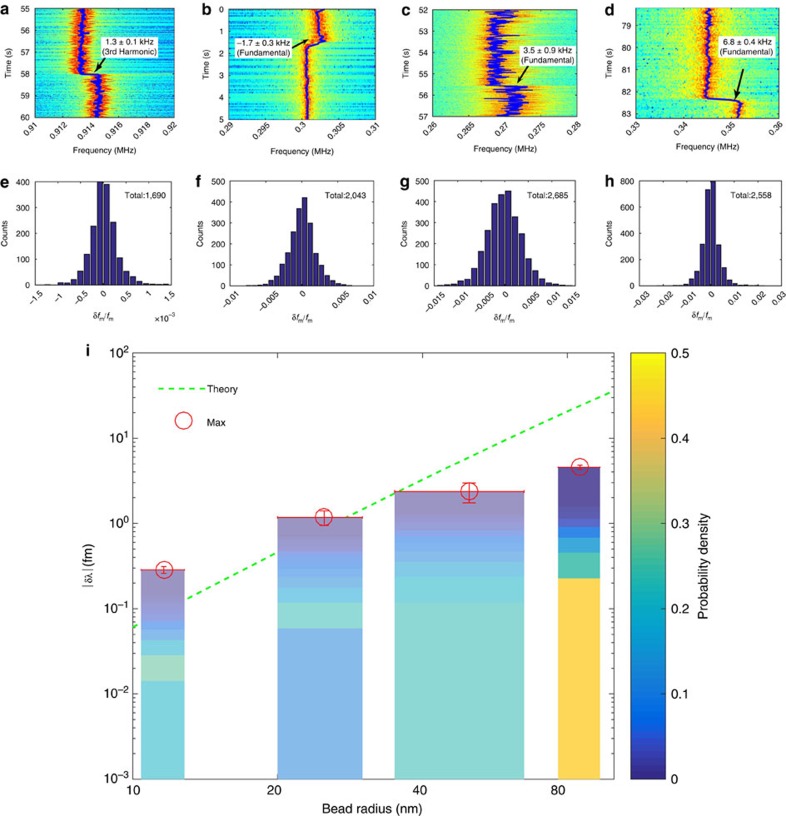
Detection of silica nanobeads. (**a**–**d**) Typical mechanical spectrograms for the binding events of silica beads with average radii of 11.6, 25, 50 and 85 nm, where **a** shows that of third harmonic and **b**–**d** show those of the fundamental oscillation frequency. The blue solid lines are the peak frequency traces computed by a least square fitting of each spectrum to a Lorentzian function. (**e**–**h**) The histograms of the normalized frequency steps *δf*_m_/*f*_m_. (**i**) The corresponding cavity resonance shifts induced by the particle binding as a function of bead radius. The colour bars show the probability density functions of the recorded cavity resonance wavelength shifts induced by particle binding, where the bar width indicates the s.d. of the bead size (provided by the manufacturer) and the colour map indicates the magnitude of probability density. The red circles indicate the recorded maximum wavelength shifts of the cavity resonance with the s.d. of the difference between the measured data and the corresponding least square fitted step function represented by error bars. The dashed curve shows the theoretical prediction^30^.

To characterize the real sensing performance, we performed the sensing experiments on silica nanobeads with different diameters. We set the laser-cavity detuning at the operational point indicated within the dashed circle of Fig. 2b and delivered silica nanobeads diluted in Dulbecco's phosphate-buffered saline (DPBS) around the microsphere. A particle binding would introduce a sudden change of the OMO frequency. One example is clearly shown in the video clip provided in Supplementary Movie 1. The particle binding events were recorded by searching for the sudden changes of the oscillation frequency in the recorded spectrograms. Typical examples are shown in Fig. 3a–d for the nanobeads with radii of 11.6, 25, 50 and 85 nm, respectively. Here in the case of 11.6-nm beads, the frequency steps were recorded at the third harmonic of the oscillation frequency while all others were obtained at the fundamental. Note the noisy spectrum of the 50-nm sensing experiment is due to the carousal trap effect reported in ref. 29, which is also evident from the video clip provided in Supplementary Movie 1. As shown in Fig. 3a, a clear step of 1.3±0.1 kHz (corresponding to 0.43±0.03 kHz step at the fundamental oscillation tone) was observed at the time of 58 s with an SNR of 13. An increase of the oscillation frequency implies a red shift of the cavity resonance wavelength, which corresponds to a binding of a 11.6-nm silica bead on the surface of the microsphere. Figure 3b–d show frequency steps of −1.7±0.3, 3.5±0.9 and 6.8±0.4 kHz, respectively, which correspond to the binding (positive frequency steps) or unbinding (negative steps) events of 25, 50 and 85 nm beads. Note that the direct contribution of particle binding to the mechanical inertia of the OMO is negligible since the masses of the nanobeads (∼0.01–5 fg depending on particle radius) are more than nine orders of magnitude smaller than the effective motional mass of the OMO (∼1 μg). The observed OMO frequency shifts induced by particle bindings are purely transduced from the optical spring effect.

To obtain the statistical properties of the binding events, we recorded a total number of 500,121, 521,389, 1,335,415 and 758,728 spectra in sensing 11.6, 25, 50 and 85 nm beads, respectively, among which frequency steps of 1,690, 2,043, 2,685 and 2,558 were captured with the SNR exceeding unity. Figure 3e–h show the histograms of the normalized frequency steps, *δf*_m_/*f*_m_, which indicate maximum OMO frequency shifts of *δf*_m_/*f*_m_=(1.4±0.4) × 10^−3^, (−7.8±1.5) × 10^−3^, (1.3±0.3) × 10^−2^ and (−2.3±0.6) × 10^−2^, respectively, for beads with radii of 11.6, 25, 50 and 85 nm. We converted the recorded OMO frequency steps into the corresponding cavity resonance wavelength shifts *δλ*, with the transduction rate of *df*_m_/*d*Δ_*λ*_=−1.5 kHz fm^−1^. The probability density function of their absolute values are plotted as colour bars in Fig. 3i, with maximum wavelength shifts of |*δλ*|/*λ*_0_=2.6 × 10^−10^, 1.2 × 10^−9^, 2.4 × 10^−9^ and 4.6 × 10^−9^, respectively, for the fourbead sizes (red circles). For comparison, we also numerically estimated the expected maximum wavelength change as a function of bead radius (green dashed line)^30^. The experimental results agree with the theoretical predictions when the bead size is small (11.6 and 25 nm). At larger bead radii (50 and 85 nm), the experimental values are smaller than the theoretical predictions because the optical *Q* starts to degrade at a large bead size. As an estimate, binding of a 85-nm bead to the equator of a 100-μm microsphere would degrade the optical *Q* by 2.7 × 10^4^. As the optical spring depends on both the laser-cavity detuning and the optical *Q*, the impact from cavity *Q* change counteracts that from the cavity resonance wavelength shift, leading to a smaller shift of OMO frequency (see Supplementary Note 1 for more details). Further discussions on nanobead results are detailed in Supplementary Notes 3 and 4.

### Detection of single protein molecules

**Figure 4:**
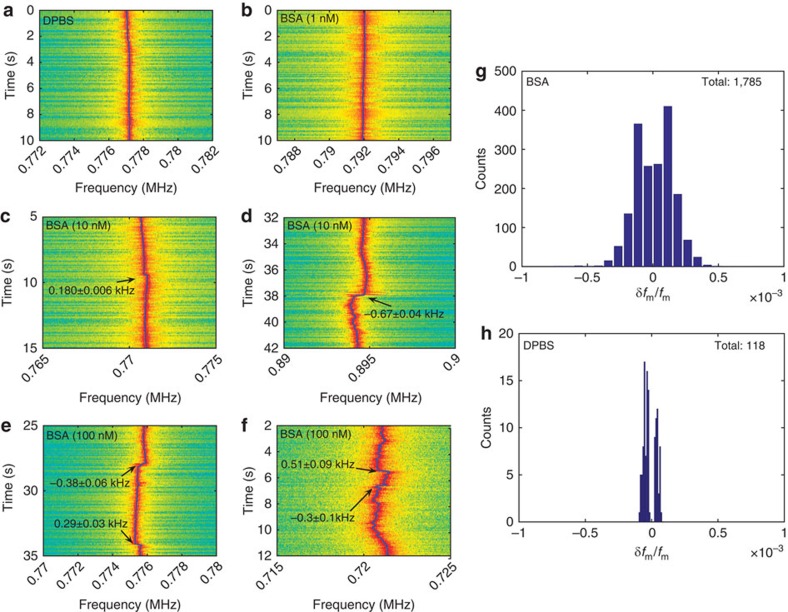
Detection of single protein molecules. (**a**–**f**) Typical mechanical spectrograms recorded at the third harmonic of the oscillation tone with different BSA concentrations. (**a**) Bare DPBS environment where protein molecules were absent. (**b**) BSA molecules with 1 nM nominal concentration were gradually injected. These two spectrograms show no binding event. (**c**,**d**) BSA molecules with 10 nM nominal concentration were injected. The spectrograms capture the event of a BSA protein molecule binding at an off-equator site at 9.4 s with a clear frequency step of 180±6 Hz (**c**), and that of a molecule detaching from the silica microsphere surface at 38 s with a clear frequency step of −670±40 Hz (**d**). (**e**,**f**) BSA molecules with 100 nM nominal concentration were injected. The spectrograms further show the corresponding binding events with increased binding frequency as well as background noises. (**g**) The histogram of the normalized frequency steps of BSA binding events. (**h**) The histogram of the normalized frequency steps in the bare DPBS environment where BSA molecules were absent.

The ultrahigh detection sensitivity demonstrated on silica nanobeads readily implies the superior capability of sensing single protein molecules. To do so, we injected BSA diluted in DPBS around the microsphere sensor, with the concentration gradually increased from 0–100 nM and plotted the typical spectrograms in Fig. 4a–f. In the protein sensing experiments, the excessive noises from the unwanted molecules were significantly reduced, resulting in a detection noise level close to the DPBS background noise obtained from the Allan deviation measurement. The third order harmonic of the oscillation frequency was employed to monitor the frequency steps. As shown in Fig. 4d, a maximum frequency step of −0.67±0.04 kHz was observed with an SNR of 16.8, which corresponds to a step of −0.22±0.01 kHz at the fundamental oscillation tone. In total, we recorded 145,407 spectra with nominal concentration up to 10 nM among which 1,785 frequency steps were captured. The histogram of the normalized frequency steps is plotted in Fig. 4g, with the maximum step of *δf*_m_/*f*_m_=(−7.6±0.4) × 10^−4^ which corresponds to a cavity resonance shift of |*δλ*|/*λ*_0_=1.5 × 10^−10^. As a comparison, we also collected spectra by immersing the microsphere in bare DPBS without any protein molecules. As shown in Fig. 4a, the spectrogram indicates the OMO is stable in the absence of BSA molecules. Further, the histogram of all 118 steps found by our programme displayed in Fig. 4h shows the baseline noise is well below the step signal detected in BSA experiments. In addition, the detailed analysis of expected frequency step size and noise level (Supplementary Note 5) further confirms that the observed signals were protein binding induced. This observation clearly proves the capability of sensing single BSA molecules with a molecular weight of 66 kDa. By assuming the resonance shift is proportional to the mass (or equivalently, to the volume) of the protein^30^, we derive that our current set-up is capable of detecting proteins as small as 3.9 kDa with an SNR above unity. A discussion of the influence of protein concentration in sensing experiments and binding time are presented in Supplementary Notes 5 and 6.

## Discussion

The demonstrated single-molecule detection now paves the foundation of ultra-sensitive cavity optomechanical spring sensing. The sensing resolution can be further improved significantly in the future. For example, the minimal detectable OMO frequency shift in current devices is primarily limited by the laser frequency jitter in our experiment. With an OMO linewidth of only ∼0.1 Hz, we expect that the future adoption of a fine laser frequency locking circuitry can further improve the sensing resolution by ∼100 times to around *δλ*/*λ*_0_∼10^−14^. On the other hand, the optical *Q* can be increased to above 10^8^ if a visible laser is employed^8^, which would further improve the sensing resolution by more than one order of magnitude. Moreover, a plasmonic structure can also be incorporated to enhance the cavity resonance shift. These future improvements would enable detecting small molecules and atoms with a mass down to sub-Dalton level in cryogenic environment, with great potential for dramatically advancing the capability of sensing to an unprecedented level.

In particular, as the molecule binding occurs during the coherent mechanical motion of the sensor, controlling the motion pattern of the coherent OMO (amplitude, phase, time waveform and so on) may function as a unique paradigm to study and control the mechanical properties of molecule binding and unbinding. This, in combination with certain functionalizations of the sensor surface^5^ and with implementation of potentially versatile optomechanical motions^19^, may offer a unique multifunctional biomolecule toolbox that is not only able to observe cellular machineries at work, but also to selectively manipulate single-molecule interactions. Moreover, although we focus here on particle and molecule sensing, the demonstrated optical spring sensing principle can be applied for other physical sensing applications^31^, such as inertial sensing^32^,^33^, electromagnetic field sensing^34^, gas sensing^35^ and so on, which are based on sensitive detection of optical cavity resonance shifts induced by external physical perturbations. Therefore, we expect the demonstrated optical spring sensing to be of great promise for broad applications beyond particle and molecule sensing itself.

## Methods

### Device fabrication and characterization

To fabricate the device, we first heated a single-mode optical fibre with a hydrogen torch and pulled it to form a tapered tip which was then reflowed into a spherical shape by a CO_2_ laser. The optical wave was coupled into and out of the device via a tapered optical fibre, as illustrated in Fig. 1a. The intrinsic optical *Q* of the device was measured on the silica microsphere immersed in DPBS (c.f. Fig. 6), with the laser wavelength calibrated by a reference interferometer. The input power was maintained low enough to prevent any optomechanical, thermo-optic or nonlinear effect. By fitting the cavity transmission spectrum with a Lorentzian function (Fig. 1c), we obtained an intrinsic optical *Q* of 4.8 × 10^6^. Its close agreement with the numerically predicted value of 6 × 10^6^ indicates a nearly complete elimination of excessive contaminants that could have otherwise degraded the optical *Q*. This also verifies that the potential impact of dangling bonds of silica, which might produce fracture on the microsphere to introduce additional loss, is negligible on optical *Q* in the regime *Q*<10^7^.

**Figure 5:**
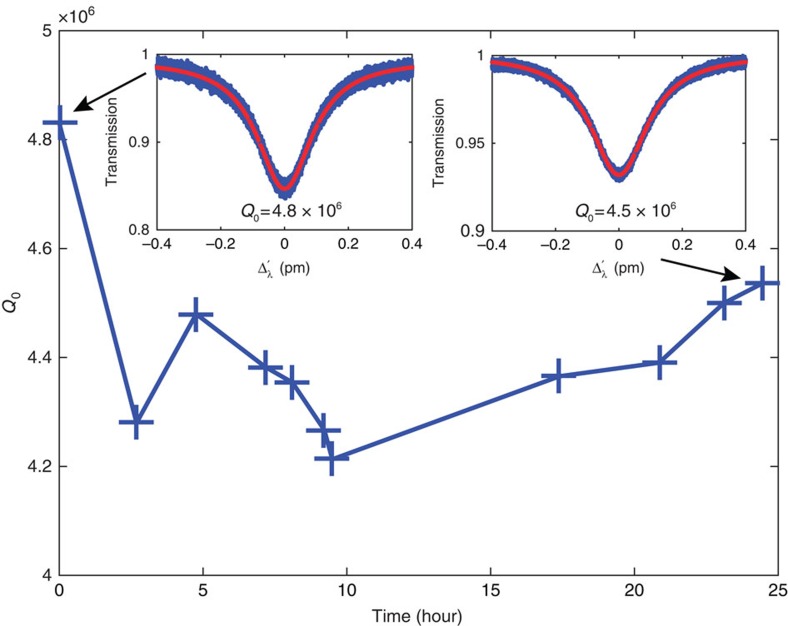
Cavity *Q* degradation test. Main plot: the intrinsic quality factor (*Q*_0_) versus time; left inset: *Q*_0_=4.8 × 10^6^ when the silica microsphere was just immersed in DPBS buffer and right inset: *Q*_0_=4.5 × 10^6^ after 25 h. The blue trace in each inset is the normalized transmission spectrum averaged over 100 traces and the red curve is the least square fit of the transmission curve to a Lorentzian function.

To further verify the long-term stability of optical *Q* of the devices after they are immersed in DPBS buffer, we monitored the intrinsic *Q* fluctuation of a silica microsphere over a day. As shown in Fig. 5, the device exhibits a high optical *Q* of 4.8 × 10^6^ right after the device was immersed in DPBS. After 25 h, the optical *Q* remains nearly intact, as high as 4.5 × 10^6^. This clearly verifies the long-term stability of optical *Q* in our devices. Note that our sensing experiments typically last no longer than 10 h, we are confident that the cavity degradation during the sensing experiments is negligible.

To excite the coherent OMO, we adjusted the spacing between the microsphere and the fibre taper to a position close to critical coupling and increased the input power to 8.5 mW. As shown in Fig. 1d, the coherent OMO started at a threshold optical power of 3.0 mW. To characterize the properties of coherent OMO, we set the wavelength of the continuous-wave laser for stable OMO and recorded the power spectrum of the signal transmitted from the cavity. The linewidth of the OMO was obtained by fitting the fundamental oscillation tone with a Lorentzian function, as shown in Fig. 1f.

### Allan deviation measurement of OMO frequency

To obtain the background noise and to determine the minimum sensing resolution of our experiment, we measured the Allan deviation (Supplementary Note 7) of the fundamental, second and third order harmonics of OMO on the device immersed in pure DPBS in the absence of particle and recorded the power spectrum of cavity transmission seamlessly. As shown in Fig. 2c, the Allan deviation reaches a minimum within a measurement interval of 256 ms with a minimum two-sample deviation of 9.5 Hz at the fundamental oscillation. This value of Allan deviation includes all background noises in the experiments, thus corresponding to the minimum detectable shift of fundamental OMO frequency with an SNR of unity. It was also observed that the Allan deviations of higher order harmonics increase almost proportionally to that of the fundamental oscillation tone, indicating that the SNR would not be improved on the higher order harmonics. However, in practice, multiple particles that skim by the cavity surface within a single spectrum acquisition time contribute to an excessive detection noise. This noise can be reduced by sensing at higher order harmonics which facilitates considerably the mechanical spectral analysis (by allowing to use a coarser resolution bandwidth and thus a faster data acquisition interval).

### Calibration of laser-cavity wavelength detuning

We set the laser wavelength at a off-resonance blue-detuned position and increased it step by step towards the cavity resonance, with a fixed input power. Both the time waveform and power spectrum of the transmitted signal were recorded at each set wavelength. The inset of Fig. 2b shows the averaged optical power injected into the cavity 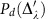 as a function of laser wavelength detuning 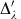, which reflects the thermo-optic bistability. The actual laser-cavity detuning, Δ_*λ*_=*λ*_*l*_−*λ*_0_ where *λ*_*l*_ is the laser wavelength, is obtained by the following equation


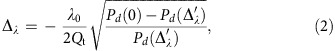


where *P*_*d*_(0) is the optical power transferred to the cavity when the probe laser is on-resonance.

### Experiment set-up

**Figure 6:**
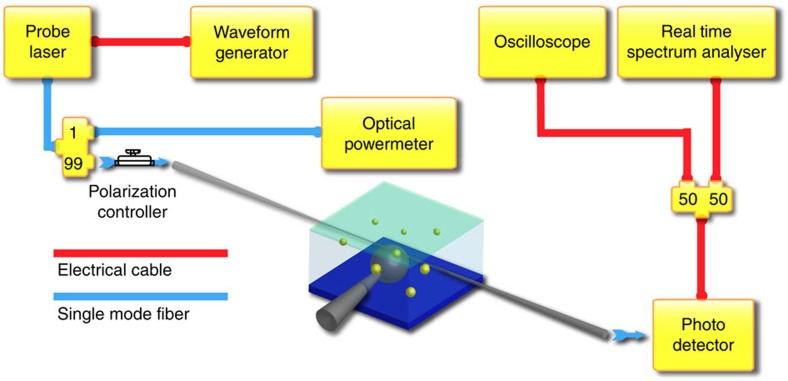
Experiment set-up. Ninety-nine per cent of the laser light is delivered to the microcavity through a tapered optical fibre. The light escaped from the cavity is coupled back to the same fibre and captured by a photodetector. The captured signal is then converted to electrical signals and analysed by a spectrum analyser and an oscilloscope. Meanwhile 1% light is delivered to an optical power metre for monitoring purposes.

The experimental set-up is illustrated in Fig. 6. Here a Newport TLB 6700 external cavity tunable laser was used as a light source. The wavelength of the laser was tuned by a waveform generator (Agilent 33210A). In our experiment, the laser output was connected to a 99/1 optical directional coupler so that 1% of the light was connected to an optical power metre (Newport 1830c) to monitor the power of the laser output. The 99% output branch was connected to a polarization controller before entering a tapered fibre. The light was then delivered to the silica microsphere through this tapered fibre while the light escaped from the microsphere was coupled to the same taper and delivered to a photodetector before converting into an electrical signal. The electrical signal was divided into two equal output ports using an electrical power splitter where one output signal from the splitter was recorded by an oscilloscope (Agilent DSO 90404A) for time domain measurements and the other output was delivered to a real time spectrum analyser (Tektronix RSA 3408B) for spectral analysis.

### Data availability

The data that support the findings of this study are available from the corresponding author on request.

## Additional information

**How to cite this article:** Yu, W. *et al.* Cavity optomechanical spring sensing of single molecules. *Nat. Commun.* 7:12311 doi: 10.1038/ncomms12311 (2016).

## Supplementary Material

Supplementary Notes and References Supplementary Notes 1-7 and Supplementary References.

Supplementary Movie 1 Optomechanical spring detection of silica nanobeads.

Peer Review File
