# Supplementary material for: Cavity optomechanical spring sensing of single molecules
Source: Nat Commun. 2016 Jul 27;7:12311. doi: 10.1038/ncomms12311 (PMC4974467; doi:10.1038/ncomms12311)
Supplement: Supplementary Notes and References — Supplementary Notes 1-7 and Supplementary References. [file ncomms12311-s1.pdf]

Supplementary Notes

Supplementary Note 1. The optomechanical effect in the sensing system

A spring pulling away from its equilibrium position will oscillate as its elasticity exerts a restoring force to counter react the mechanical deformation. In a whispering gallery microcavity, the circulating optical wave exerts an optical force to deform the cavity from its equilibrium, causing it to oscillate mechanically in the same manner as a spring. Meanwhile, the cavity resonance wavelength changes as a result of the mechanical deformation, which in turn modulates the light intensity and the optical gradient force. The mutual opto-mechanical coupling in the process makes the cavity a forced harmonic oscillator and this phenomenon is also known as optomechanical oscillation (OMO)[1–6]. It is well known that the harmonic oscillation angular frequency Ω_m of a spring follows the Hooke's law $\Omega_m = \sqrt{\frac{k}{m_{eff}}}$ where m_{eff} is the effective motional mass of a particular mechanical oscillation mode and k the spring constant. In analogy, the oscillation angular frequency of the OMO can be described by the same equation, by including the radiation pressure contribution to the spring constant, $k = k_{mech} + k_{opt}$. Here, k_{mech} is the cavity elasticity induced intrinsic mechanical spring constant and k_{opt} is the optical spring constant produced by the optomechanical coupling. As the magnitude of the radiation pressure changes due to the cavity resonance detuning, the optical spring constant depends sensitively on the cavity resonance. Such an optomechanical effect is described in detail in the following.

The whispering-gallery optical wave (Fig. 1a of the main text) produces a radiation pressure that drives the radial breathing mechanical mode of a microsphere. The mechanical mode for a microsphere sitting on a post (Fig. 1b of the main text) has a motion that modulates the device radius (Fig. 1a of the main text) which in turn modulates the optical cavity resonance. Such an optomechanical coupling is described by the following coupled equations of motion[1]:

$$\frac{da}{dt} = \left(i\Delta_\omega - \frac{\Gamma_t}{2} - ig_{om}x\right)a + i\sqrt{\Gamma_e}A_{in}, \tag{1}$$

$$\frac{d^2x}{dt^2} + \Gamma_m\frac{dx}{dt} + \Omega_m^2 x = \frac{F_{rad}(t)}{m_{eff}} + \frac{F_L(t)}{m_{eff}}, \tag{2}$$

where a is the field amplitude of optical whispering-gallery mode (WGM), normalized such that $U = |a|^2$ represents the cavity mode energy. A_{in} is the input optical wave, normalized such that $P_{in} = |A_{in}|^2$ represents the input optical power. Γ_t is the photon decay rate of the loaded cavity, and Γ_e is the photon decay rate associated with the external waveguide coupling. $\Delta_\omega = \omega_l - \omega_0$ is the

frequency detuning from the input wave ω_l to the cavity resonance ω_0. x represents the effective mechanical displacement of the radial breathing mode of the microsphere. $\Gamma_{\rm m}$, $\Omega_{\rm m}$, and $m_{\rm eff}$ are the damping rate, intrinsic resonance frequency, and effective mass of the mechanical mode, respectively. $g_{\rm om} = \frac{d\omega_0}{dx}$ is the optomechanical coupling coefficient, which scales inversely with the radius for a microsphere, with a magnitude of $|g_{\rm om}|/(2\pi) \approx 6.2$ GHz nm^{-1} at a wavelength of 974 nm for a device with a diameter of 100 μm. $F_{\rm rad} = -\frac{g_{\rm om}|a|^2}{\omega_0}$ describes the radiation pressure produced by the optical wave inside the cavity and $F_{\rm L}$ is the thermal Langevin force responsible for the thermal Brownian motion of the mechanical mode.

For a continuous-wave (CW) laser input with a constant power, the backaction between the intracavity optical wave and the mechanical motion modifies the dynamics of the mechanical mode, resulting in an effective mechanical damping rate $\Gamma'_{\rm m}$ and an effective mechanical frequency $\Omega'_{\rm m}$ given by[1],

$$\Gamma'_{\rm m} \approx \Gamma_{\rm m} - \frac{2g_{\rm om}^2 P_{\rm in}}{m_{\rm eff}\omega_0} \frac{\Delta_\omega \Gamma_{\rm t}\Gamma_{\rm e}}{(\Gamma_{\rm t}/2)^2 + \Delta_\omega^2} \frac{1}{[(\Delta_\omega + \Omega_{\rm m})^2 + (\Gamma_{\rm t}/2)^2][(\Delta_\omega - \Omega_{\rm m})^2 + (\Gamma_{\rm t}/2)^2]}, \qquad (3)$$

$$\Omega'^{\,2}_{\rm m} \approx \Omega_{\rm m}^2 + \frac{2g_{\rm om}^2 P_{\rm in}}{m_{\rm eff}\omega_0} \frac{\Delta_\omega \Gamma_{\rm e}}{(\Gamma_{\rm t}/2)^2 + \Delta_\omega^2} \frac{\Delta_\omega^2 - \Omega_{\rm m}^2 + (\Gamma_{\rm t}/2)^2}{[(\Delta_\omega + \Omega_{\rm m})^2 + (\Gamma_{\rm t}/2)^2][(\Delta_\omega - \Omega_{\rm m})^2 + (\Gamma_{\rm t}/2)^2]}, \qquad (4)$$

which are obtained from Eq. (1) and (2) by treating the mechanical motion as a perturbation to the optomechanical system.

Equation (3) shows the effect of optomechanical amplification/cooling, depending on the laser-cavity detuning. Physically, the CW optical wave launched into the cavity produces radiation pressure around the equator of the microsphere along the radial direction to actuate the radial-breathing mechanical mode (Eq. (2)). The induced mechanical motion changes the cavity length and thus shifts the optical resonance frequency, which in turn modulates the optical wave inside the cavity (Eq. (1)). The resulting dynamic backaction between the optical field and mechanical motion can be either in phase or out of phase with the mechanical motion, depending on the laser-cavity detuning, thus amplifying or cooling the mechanical motion. The efficiency of optomechanical amplificaiton/cooling depends on the optomechanical coupling strength $g_{\rm om}$, the optical Q, and the laser-cavity detuning Δ_ω. Therefore, the high optical quality and strong optomechanical coupling in our device would provide efficient optomechanical excitation.

On the blue detuning side ($\Delta_\omega > 0$), the optical wave amplifies the mechanical motion and leads to a decrease of the mechanical damping rate that depends linearly on the optical power. A large enough optical power is able to boost the mechanical motion above the oscillation threshold,

as shown in Fig. 1d-f of the main text, resulting in a coherent optomechanical oscillation (OMO) with a very narrow linewidth. Equation (4) shows the optical spring effect where the mechanical frequency depends sensitively on the laser-cavity detuning Δ_ω. This effect underlies the principle of cavity optomechanical spring sensing, which will be discussed in detail in the following.

Equation (4) indicates that a maximum mechanical frequency $\Omega'_{\rm m}$ occurs at $\Delta_\omega^{max} = \frac{\Gamma_{\rm t}}{2\sqrt{3}}$ which corresponds to a laser-cavity wavelength detuning of $\Delta_\lambda^{max} = -\frac{\lambda_0}{2\sqrt{3}Q_{\rm t}}$. In our experiment, Δ_λ^{max} is measured to be -119 fm (Fig. 2b of the main text), corresponding to a loaded optical Q of $Q_{\rm t} \approx 2.4\times10^6$. In an aqueous environment, the viscosity of the surrounding fluid significantly impacts the mechanical motion of the device, resulting in a dramatically damped mechanical mode with very low mechanical frequency. The mechanical motion is so heavily damped that the intrinsic thermal Brownian motion cannot be clearly detected. As the numerical modeling of the fluidic system is beyond the capability of our current numerical modeling software, we treat the effective mass and the intrinsic mechanical frequency as free parameters in our theoretical analysis. We calculate the mechanical frequency as a function of laser-cavity detuning, which is plotted as a gray solid line in Fig. 2b of the main text. It agrees closely with the experimental observations, with a slight discrepancy likely because of the significant amplitude of coherent OMO (Fig. 1d of the main text) that is beyond the linear perturbation regime used in the theory. Figure 2b of the main text shows that the effective rigidity of the mechanical mode is dominated by the optically induced spring in the device and the intrinsic mechanical rigidity plays a fairly minor role.

In general, particle binding on the microcavity modifies both the optical cavity resonance and the optical Q. Equation (4) shows that the mechanical frequency depends on both the laser-cavity detuning and the optical Q, yielding a shift of the OMO frequency given by

$$\delta\Omega'_{\rm m} = \frac{\partial\Omega'_{\rm m}}{\partial\omega_0}\delta\omega_0 + \frac{\partial\Omega'_{\rm m}}{\partial Q_{\rm t}}\delta Q_{\rm t} = -\frac{\partial\Omega'_{\rm m}}{\partial\Delta_\omega}\delta\omega_0 - \frac{\Gamma_{\rm t}}{Q_{\rm t}}\frac{\partial\Omega'_{\rm m}}{\partial\Gamma_{\rm t}}\delta Q_{\rm t}, \tag{5}$$

where $\delta\omega_0$ and $\delta Q_{\rm t}$ are the variations of cavity resonance frequency and loaded cavity optical Q, respectively, induced by the particle binding. From Eq. (4), we obtain

$$\frac{\partial\Omega'_{\rm m}}{\partial\Delta_\omega} = \frac{g_{\rm om}^2 P_{\rm in}\Gamma_{\rm e}}{m_{\rm eff}\omega_0\Omega'_{\rm m}L_0L_+L_-}\left[L_0 + \Omega_{\rm m}^2\left(\frac{2\Delta_\omega^2}{L_0} - 1\right) - \frac{4\Delta_\omega^2(L_0-\Omega_{\rm m}^2)^2}{L_+L_-}\right], \tag{6}$$

$$\frac{\partial\Omega'_{\rm m}}{\partial\Gamma_{\rm t}} = \frac{g_{\rm om}^2 P_{\rm in}\Gamma_{\rm e}\Gamma_{\rm t}\Delta_\omega}{2m_{\rm eff}\omega_0\Omega'_{\rm m}L_0L_+L_-}\left[1-(L_0-\Omega_{\rm m}^2)\left(\frac{1}{L_0}+\frac{1}{L_+}+\frac{1}{L_-}\right)\right], \tag{7}$$

where $L_0 \equiv \Delta_\omega^2 + (\Gamma_{\rm t}/2)^2$, $L_+ \equiv (\Delta_\omega+\Omega_{\rm m})^2 + (\Gamma_{\rm t}/2)^2$, and $L_- \equiv (\Delta_\omega-\Omega_{\rm m})^2 + (\Gamma_{\rm t}/2)^2$.

In the sideband-unresolved regime with $\Omega_{\rm m} \ll \Gamma_{\rm t}$ where our sensing experiments operates,

Eqs. (6) and (7) can be simplified considerably to

$$\frac{\partial \Omega'_{\rm m}}{\partial \Delta_\omega} = \frac{g_{\rm om}^2 P_{\rm in}\Gamma_{\rm e}}{m_{\rm eff}\omega_0\Omega'_{\rm m}} \frac{(\Gamma_{\rm t}/2)^2 - 3\Delta_\omega^2}{[\Delta_\omega^2 + (\Gamma_{\rm t}/2)^2]^3}, \quad (8)$$

$$\frac{\partial \Omega'_{\rm m}}{\partial \Gamma_{\rm t}} = -\frac{g_{\rm om}^2 P_{\rm in}\Gamma_{\rm e}}{m_{\rm eff}\omega_0\Omega'_{\rm m}} \frac{\Gamma_{\rm t}\Delta_\omega}{[\Delta_\omega^2 + (\Gamma_{\rm t}/2)^2]^3}. \quad (9)$$

When the particle size is small, the binding events introduce negligible impact on the optical Q, resulting in a linear relationship between OMO frequency shift and the optical cavity resonance shift, as shown in Eq. (5). In the cases when the particle size is large enough to yield a substantial degradation of the cavity Q, Eq. (5) indicates a deviation from such linear dependence due to the contribution of the δQ term at the sensing operational regime. This agrees with the experimental observations shown in Fig. 3c of the main text.

The discussions above show that the OMO frequency depends linearly on the laser-cavity detuning, providing that particle/molecule binding events introduce negligible cavity optical Q degradation. This mechanism offers an elegant approach to probe particle/molecule optomechanically. As the minimal detectable OMO frequency shift is determined by the linewidth of coherent OMO, from Eqs. (5) and (8), we obtain the sensing resolution given by Equation (1) of the main text, with the optomechanical transduction factor given by

$$\eta_{\rm om} = \left(1 - \frac{\Omega_{\rm m}^2}{\Omega_{\rm m}'^2}\right) \frac{1 - 3\bar{\Delta}_\omega^2}{(1+\bar{\Delta}_\omega^2)\bar{\Delta}_\omega}, \quad (10)$$

where $\bar{\Delta}_\omega = \Delta_\omega/(\frac{\Gamma_{\rm t}}{2})$ is the normalized detuning. For our sensing system, the OMO frequency is dominated by the optical spring, $\Omega'_{\rm m} \gg \Omega_{\rm m}$. As a result, the value of $\eta_{\rm om}$ primarily depends on the laser-cavity detuning and it usually ranges from 1 to 2 for proper sensing operation. At the present operation point with $\bar{\Delta}_\omega \approx 0.35$ used in the experiment, $\eta_{\rm om} \approx 1.6$.

Supplementary Note 2. Distinction from conventional optomechanical and nanomechanical sensing

Here we provide a simple picture to show the distinctive nature of our proposed approach compared with conventional optomechanical and nanomechanical sensing. The oscillation angular frequency of the OMO is given simply by the Hooke's law, $\Omega'_m = \sqrt{\frac{k}{m_{\rm eff}}}$, where $m_{\rm eff}$ is the effective motional mass and k is the effective spring constant. As discussed in the previous section, the spring constant of an OMO contributes from two parts, $k = k_{\rm mech} + k_{\rm opt}$, where $k_{\rm mech}$ comes from the intrinsic mechanical rigidity of the device structure and $k_{\rm opt}$ is the optically induced spring

which can be obtained from Eq. (4). Consequently, the OMO frequency shift can be induced by perturbation either to the mass δm_{eff}, or to the spring constant, δk, given below:

$$\frac{\delta\Omega_m'}{\Omega_m'} = \frac{1}{2}\left(\frac{\delta k}{k} - \frac{\delta m_{\text{eff}}}{m_{\text{eff}}}\right) = \frac{1}{2}\left(\frac{\delta k_{\text{opt}}}{k} + \frac{\delta k_{\text{mech}}}{k} - \frac{\delta m_{\text{eff}}}{m_{\text{eff}}}\right). \tag{11}$$

Conventional nanomechanical sensing relies on the last two terms, $\frac{\delta\Omega_m'}{\Omega_m'} = \frac{1}{2}\left(\frac{\delta k_{\text{mech}}}{k} - \frac{\delta m_{\text{eff}}}{m_{\text{eff}}}\right)$, where the molecule binding either perturbs the effective mass or modifies the intrinsic mechanical spring constant of the mechanical resonator[7,8]. Conventional OMO sensing relies on the last term, $\frac{\delta\Omega_m'}{\Omega_m'} = -\frac{1}{2}\frac{\delta m_{\text{eff}}}{m_{\text{eff}}}$, similar to nanomechanical sensing while with an optical readout[6,9–11].

In contrast, our approach takes advantage of the first term, $\frac{\delta\Omega_m'}{\Omega_m'} = \frac{1}{2}\frac{\delta k_{\text{opt}}}{k}$. In particular, cavity optomechanics produces an optically induced spring that depends sensitively on the laser-cavity detuning, $\delta k_{\text{opt}} = \frac{dk_{\text{opt}}}{d\omega_0}\delta\omega_0$, where $\delta\omega_0$ is the resonance shift of the optical cavity induced by the molecule binding. Consequently, the OMO frequency shift is given by (see also Eq. (5))

$$\frac{\delta\Omega_m'}{\Omega_m'} = \frac{1}{2k}\frac{dk_{\text{opt}}}{d\omega_0}\delta\omega_0. \tag{12}$$

As discussed in the previous section, cavity optomechanics results in a significant magnitude of frequency tuning slope, $\frac{dk_{\text{opt}}}{d\omega_0}$, that dramatically amplifies the cavity resonance shift induced by molecular binding and transduces it into the OMO frequency shift. It is this very distinctive nature of optical spring that provides a sensing resolution orders of magnitude higher than conventional OMO or nanomechanical sensing.

Supplementary Note 3. Comments on the 50-nm-radius nanobeads spectrogram

The spectrogram of 50-nm-radius sensing experiment displayed larger background noise as illustrated in Fig. 3c. We believe that at this bead size, the optical force is sufficient to push the beads orbiting along the equator[12], which produces noisy mechanical spectrum with a magnitude similar to the binding induced step size. This is clearly evident in the video provided in the Supplementary Information.

Supplementary Note 4. Thermal-optic locking

The laser-cavity detuning is fixed primarily by the thermal-optic locking mechanism[13], which helps reduce the low-frequency perturbation from thermal/mechanical noise and laser frequency

noises[14]. The total contribution of various noise sources is reflected in the Allan deviation measurement, which is about 9 Hz on the frequency variation of optomechanical oscillation. The small value of the Allan deviation measurement indicates the high stability of our system and the high resolution of our sensing approach.

Supplementary Note 5. BSA detection at different nominal concentrations

To further confirm our protein sensing results, we did measurements at different nominal protein concentrations from 0 to 100 nM. The typical results are shown in Fig. 4a-f of the main text. The spectrograms were collected at different experiments except that Fig. 4b, 4c and 4e of the main text were measured on the same microsphere accommodated with reduced measurement duration. Note that, due to the combined dilution and diffusion effects, the actual protein concentration were typically lower than the nominal concentration. In particular, the actual protein concentrations of the three figures collected from the same microsphere were substantially lower than the nominal concentrations due to the further reduction of measurement time at each concentration. As seen, at 0 nM (DPBS only), no steps were found (Fig. 4a of the main text), neither did we find any steps at a nominal concentration of 1 nM as illustrated in Fig. 4b. At a nominal concentration of 10 nM, binding events became visible and more frequent within the measurement period (Fig. 4c and 4d of the main text). In Fig. 4c, an off-equator binding event were observed at 9.44 second with a frequency step of 180$\pm$6 Hz. The low measurement uncertainty of 6 Hz confirms that the actual protein concentration around the microsphere when the binding occurred was substantially lower than the nominal 10 nM concentration. In a separate experiment, when the measurement time is much longer so that the actual concentration is closer to 10 nM, a maximum step of -0.67 ± 0.04 kHz, inferring a molecule detaching from the microsphere equator. The increased measurement uncertainty of 40 Hz further confirms the rising of excessive noises from the increasing number of protein molecules close to the cavity when the actual protein concentration is closer to 10 nM. At a nominal 100 nM concentration, a step of -0.38 ± 0.06 kHz was found followed by another step of 0.29$\pm$0.03 kHz (Fig. 4e of the main text). Again the increasing binding frequency was observed and the increasing background noise from Fig. 4c of the main text(conducted on the same device) was obvious. In another typical experiment when the actual concentration was close to 100 nM as shown in Fig. 4f of the main text, the further increase of frequency steps and background noises was observed, indicating a substantial number of protein

molecules were available around the cavity. Due to the increasing excessive noise and the possibility of double binding events at 100 nM, our histogram (Fig.4h) only include the frequency steps occurred at concentrations up to 10 nM.

In addition to the substantial difference between the histograms of BSA and DPBS displayed in Fig. 4g and 4h of the main text, the strong correlation between actual protein concentration around the cavity and the step signal properties (noise and binding frequency) further confirms that the steps we captured in the experiments were protein binding induced.

Supplementary Note 6. Particle binding time

The time for a single particle to reach the proximity of the cavity equator highly depends on the parameters such as particle size, concentration and the distance from the sample injection point to the cavity surface. In our experiments, we gradually increased the sample concentration from low to high. This further complicated the accurate estimation of the particle binding time. In general, by injecting a small portion of 100 nM BSA (about 10 % of the liquid in the sample cell) from a distance of around 0.2 cm, it took approximately 15 minutes for a binding event to be detected. Faster binding time is possible by increasing the sample concentration and delivering it at a closer location to the microsphere.

Supplementary Note 7. Allan deviation

Allan deviation[15], named after David W. Allan, is a powerful technique to characterize the frequency stability of an oscillator where many kinds of noises such as flicker noises, random walk noises may lead to a divergent value of the classical standard deviation. For oscillation frequency signals measured continuously in a time interval T, the Allan deviation $\sigma(\tau)$ can be expressed as

$$\sigma(\tau) = \sqrt{\frac{1}{2}\langle (f_m(t+\tau) - f_m(t))^2 \rangle_t} \tag{13}$$

Here $f_m(t)$ is the oscillation frequency measured at time t and $\langle(\cdot)\rangle_t \equiv \frac{1}{T}\int_0^T(\cdot)dt$ denotes the time average of the quantity $(\cdot)$ over T. When a fully incoherent noise such as white noise is the only dominant noise, the Allan deviation is identical to the standard deviation. As f_m is usually measured at discrete time, the above equation needs to be discretized. In our experiment, the OMO frequencies were measured by an realtime electrical spectrum analyzer with a preset sampling rate of δt per spectrum. By representing the measured N OMO frequencies as a time series

$f_m(0), f_m(\delta t), f_m(2\delta t), \ldots, f_m((N-1)\delta t)$, the Allan deviation at $\tau = m\delta t, (m = 1, 2, \ldots, N)$ can be derived as

$$\sigma(\tau = m\delta t) = \sqrt{\frac{1}{2(N-m)} \sum_{k=0}^{N-m-1} (f_m((k+m)\delta t) - f_m(k\delta t))^2} \quad (14)$$

It is worth mentioning that the Allan deviation typically displays a minimum at τ_{min}. Consequently we can set the acquisition time interval of our spectrum analyzer close to τ_{min} to minimize the measurement noise. Note that in our experiment, τ_{min} is found to be around 0.1 second, in close agreement with previously reported results[16,17].

Supplementary References

1. Aspelmeyer, M., Kippenberg, T. J. & Marquardt, F. Cavity optomechanics. *Rev. Mod. Phys.* **86**, 1391–1452 (2014).
2. Thourhout, D. V. & Roels, J. Optomechanical device actuation through the optical gradient force. *Nat. Photonics* **4**, 211–217 (2010).
3. Favero, I. & Karrai, K. Optomechanics of deformable optical cavities. *Nat. Photonics* **3**, 201–205 (2009).
4. Marquardt, F. & Girvin, S. M. Optomechanics. *Physics* **2**, 40:1–7 (2009).
5. Müller, D. J. & Dufrene, Y. F. Atomic force microscopy as a multifunctional molecular toolbox in nanobiotechnology. *Nat. Nanotechnol.* **3**, 261–269 (2008).
6. Forstner, S. *et al.* Cavity optomechanical magnetometer. *Phys. Rev. Lett.* **108**, 120801:1–5 (2012).
7. Waggoner, P. S. & Craighead, H. G. Micro- and nanomechanical sensors for envinronmental, chemical, and biological detection. *Lab on a Chip* **7**, 1238–1255 (2007).
8. Arlett, J. L., Myers, E. B. & Roukes, M. L. Comparative advantages of mechanical biosensors. *Nat. Nanotechnol.* **6**, 203–215 (2011).
9. Liu, F. & Hossein-Zadeh, M. Mass sensing with optomechanical oscillation. *Sensors Journal, IEEE* **13**, 146–147 (2013).
10. Kim, K. H. & Fan, X. Surface sensitive microfluidic optomechanical ring resonator sensors. *Appl. Phys. Lett.* **105**, 191101:1–4 (2014).

[11] Liu, F., Alaie, S., Leseman, Z. C. & Hossein-Zadeh, M. Sub-pg mass sensing and measurement with an optomechanical oscillator. *Opt. Express* **21**, 19555–19567 (2013).

[12] Keng, T. K. D. *Whispering Gallery Mode Bioparticle Sensing and Transport*. Ph.D. thesis, Polytechnic Institute of New York University (2009).

[13] Carmon, T., Yang, L. & Vahala, K. Dynamical thermal behavior and thermal self-stability of microcavities. *Opt. Express* **12**, 4742–4750 (2004).

[14] Li, J., Diddams, S. & Vahala, K. J. Pump frequency noise coupling into a microcavity by thermo-optic locking. *Opt. Express* **22**, 14559–14567 (2014).

[15] Allan, D. W., Ashby, N. & Hodge, C. C. *The Science of TimekeeTime, Hewlett Packard Application Note 1289* (1997).

[16] Olcum, S. *et al.* Weighing nanoparticles in solution at the attogram scale. *Proc. Natl Acad. Sci. USA* **111**, 1310–1315 (2014).

[17] Hyun Kim, K. *et al.* Cavity optomechanics on a microfluidic resonator with water and viscous liquids. *Light: Science & Applications* **2**, e110:1–5 (2013).
