# Supplementary material for: Cavity optomechanical spring sensing of single molecules
Source: Nat Commun. 2016 Jul 27;7:12311. doi: 10.1038/ncomms12311 (PMC4974467; doi:10.1038/ncomms12311)
Supplement: Peer Review File [file ncomms12311-s3.pdf]

Editorial Note: this manuscript has been previously reviewed at another journal that is not operating a transparent peer review scheme. This document only contains reviewer comments and rebuttal letters for versions considered at Nature Communications.

Reviewers' comments:

Reviewer #3 (Remarks to the Author):

As the previous reviewer #3, I find that all of my comments have been answered with great clarity in the manuscript revision.

To repeat

"I believe that this novel method for ultra-sensitive single nano-particle and single molecule detection should be of great interest to readers of Nature Nanotechnology."

2. Response to Reviewer #2's comments

[Comment 2-1] *As the previous reviewer #3, I find that all of my comments have been answered with great clarity in the manuscript revision.*

To repeat

"I believe that this novel method for ultra-sensitive single nano-particle and single molecule detection should be of great interest to readers of Nature Nanotechnology."

[RE 2-1] We thank the reviewer's encouraging comments to our manuscript and it's our pleasure to contribute to our community a powerful tool for single molecule detection.
